# Health-related quality of life and return to work 1 year after major trauma from a network perspective

**DOI:** 10.1007/s00068-021-01781-2

**Published:** 2021-09-12

**Authors:** Jan C. van Ditshuizen, Esther M. M. van Lieshout, Ed F. van Beeck, Michiel H. J. Verhofstad, Dennis den Hartog, Dutch Trauma Registry Southwest, N. M. R. Soesman, T. S. C. Jakma, M. Waleboer, M. Staarink, M. M. M. Bruijninckx, A. Y. M. V. P. Cardon, P. T. den Hoed, G. R. Roukema, C. H. van der Vlies, N. W. L. Schep, L. van de Schoot

**Affiliations:** 1 Trauma Research Unit, Department of Surgery, Erasmus MC, University Medical Center Rotterdam, P.O. Box 2040, 3000 CA Rotterdam, The Netherlands; 2 Department of Public Health, Erasmus MC, University Medical Center Rotterdam, P.O. Box 2040, 3000 CA Rotterdam, The Netherlands

**Keywords:** Trauma Registry, Major trauma, EQ-5D-5L, Cognition, Return to work

## Abstract

**Introduction:**

Major trauma often results in long-term disabilities. The aim of this study was to assess health-related quality of life, cognition, and return to work 1 year after major trauma from a trauma network perspective.

**Methods:**

All major trauma patients in 2016 (Injury Severity Score > 15, *n* = 536) were selected from trauma region Southwest Netherlands. Eligible patients (*n* = 365) were sent questionnaires with the EQ-5D-5L and questions on cognition, level of education, comorbidities, and resumption of paid work 1 year after trauma.

**Results:**

A 50% (*n* = 182) response rate was obtained. EQ-US and EQ-VAS scored a median (IQR) of 0.81 (0.62–0.89) and 70 (60–80), respectively. Limitations were prevalent in all health dimensions of the EQ-5D-5L; 90 (50%) responders reported problems with mobility, 36 (20%) responders reported problems with self-care, 108 (61%) responders reported problems during daily activities, 129 (73%) responders reported pain or discomfort, 70 (39%) responders reported problems with anxiety or depression, and 102 (61%) of the patients reported problems with cognition. Return to work rate was 68% (37% full, 31% partial). A median (IQR) EQ-US of 0.89 (0.82–1.00) and EQ-VAS of 80 (70–90) were scored for fully working responders; 0.77 (0.66–0.85, *p* < 0.001) and 70 (62–80, *p* = 0.001) for partial working respondents; and 0.49 (0.23–0.69, *p* < 0.001) and 55 (40–72, *p* < 0.001) for unemployed respondents.

**Conclusion:**

The majority experience problems in all health domains of the EQ-5D-5L and cognition. Return to work status was associated with all health domains of the EQ-5D-5L and cognition.

## Introduction

In the global burden of disease, trauma is a major contributor to death and disabilities [1]. Surviving trauma can trigger a variety of problems in daily life. Distress and disabilities can have a profound impact on the well-being of the individual, as well as on their social network [2]. Trauma also gives rise to high societal costs due to health care dependence and a partial or complete inability to work [3].

In the past decades, trauma networks have been implemented. Such networks help to improve outcome of trauma care [4, 5]. Primary focus of these networks has long been improving survival. Since mortality has substantially been reduced, focus in trauma care has been broadened toward long-term functioning after trauma from a functional and psychological perspective [6, 7].

Over the past 2 decades, many studies have been conducted on (health-related) quality of life (QoL) after major trauma (MT, ISS > 15). These publications mainly focus on MT patients admitted to designated (major) Trauma Centers (TC). However, many MT patients also being admitted to non-Trauma Centers (NTC) [8]. Only a few cohorts have followed patients from a trauma network perspective [7, 9, 10].

Most trauma registries identify the severity of injuries with the Injury Severity Score (ISS) [11]. which is derived from the Abbreviated Injury Scale (AIS) [12]. AIS revisions better reflect contemporary state of the art of trauma care. A few QoL studies focusing on MT have used more recent AIS revisions for calculating ISS in their cohort [10, 13, 14].

EuroQoL 5D-3L is predominantly used as an instrument to measure QoL in the previous studies on MT. Since EQ-5D-5L was introduced, more differentiation is possible in distinguishing between minor levels of impairment. QoL studies focusing on MT that have used EQ-5D-5L in combination with injury coding using recent AIS revision are scarce [13].

The aim of this study was to assess health-related quality of life, cognition, and return to work (RTW) 1 year after MT from a regional trauma network perspective.

## Methods

The local Medical Research Ethics Committee exempted this study. Following review of the protocol, they concluded that the study is not subject to the Medical Research Involving Human Subjects Act (MEC-2017-041).

### General setting and data collection

Trauma region Southwest Netherlands is a large and diverse trauma region, with a level I TC, 11 level II/III NTC hospitals, a Burn Center, a dedicated Eye Hospital, and three Emergency Medical Services (EMS). It consists of rural, remote, industrial, urban, and touristic areas with a dense infrastructure inhabited by 2.5 million people. It has an even larger catchment area with the availability of Helicopter Emergency Medical Services (HEMS).

Trauma region Southwest Netherlands participates in the Dutch National Trauma Registry (DNTR) [15] with the Dutch Trauma Registry Southwest (DTR SW) cohort. All major trauma patients (ISS > 15) admitted to an emergency department within 48 h after trauma following either hospital admission, secondary transfer, or death were included, excluding death on arrival, double registries due to secondary transfers, death within the first year after trauma, or residency abroad. Injuries were coded with AIS revision 2005 update 2008 (AIS08) [16]. All included MT patients were retrospectively selected from the DNTR. One year after trauma, data from the DTR SW on demographics, injury coding, prehospital setting, and clinical outcome measures of the 2016 MT cohort were linked with the results of a questionnaire containing the EQ-5D-5L, questions on cognition, return to (paid) work (RTW), and overall health state compared with pre-injury status (three-level answer options nothing changed, worse, or better), making it a mixed-methods study.

All 12 DTR SW hospitals participated in this study. One year after trauma, the municipal base administration was checked whether or not patients had died. Each week, included patient injured 12 months prior were contacted. Non-fatally injured patients living in The Netherlands or the Flemish region of Belgium were sent a patient information letter, an informed consent form, and a questionnaire in Dutch. All questionnaires were self-reported, but respondents were allowed to ask for help. If patients lived in the Netherlands but had a language barrier, they were advised to complete the questionnaire with a relative that could help linguistically. For children younger than 13, it was compulsory to complete the questionnaire and consent together with their parents or legal guardian. Children between 13 and 18 years of age were obliged to include consent of a parent or other guardians and allowed to complete the questionnaire themselves. If adult patients were incapacitated, proxies were allowed to sign informed consent. Patients who did not respond after 1 month were contacted by telephone until contact. All questionnaires contained the adult version of the EQ-5D-5L.

### Responder and non-responders

To determine whether or not the responders were a representative sample of the population, demographic, injury-related parameters, as well as the probability of survival, were compared between responders and non-responders.

### Comorbidity and education

Comorbidities were surveyed with a modified version of the Cumulative Illness Rating Scale (CIRS), which is validated as an indicator of health status [17].

Educational level was trichotomised in ‘low’ (no diploma, primary school, or secondary vocational education), ‘middle’ (senior secondary or general vocational education, or university preparatory education), and ‘high’ (university of applied science or academic).

### EuroQol-5D-5L and cognition

Health-related QoL was measured by the EQ-5D-5L which is a valid instrument for measuring QoL in healthy, chronically ill, and trauma populations [18–20]. It consists of two summary parts: EQ-US and EQ-VAS. EQ-US is a summed score over five health dimensions that are scored with five levels (‘no problems’, ‘slight problems’, ‘moderate’, ‘severe’, and ‘unable). Health dimensions are mobility, self-care, daily activities, pain or discomfort, and anxiety or depression. Each scored health dimension adds a utility score to a summary score (range − 0.33 to 1.00, higher scores represent better QoL). With the EQ-VAS, an overall health state is scored on a Visual Analogue Scale from the worst imaginable health state to the best imaginable health state (0–100). Since the EQ-5D-5L does not capture cognitive functioning, an extra question was added and considered as a sixth health dimension with the same alteration of the abovementioned five-level answer options as used in the EQ-5D [21–23]. All health dimensions of the EQ-5D-5L and cognition were dichotomised in ‘no limitations’ (‘no problems’) and ‘limitations’ (from ‘slight problems’ to ‘unable’). Subgroup analysis on EQ-US and EQ-VAS were done for gender, age, type of injury, ISS, and Maximum AIS (MAIS, most severe AIS code).

### Return to work

Working age population was considered to be 18–65 years. Patients were asked how many days and how many hours per week they had paid work before and 1 year after trauma. RTW was trichotomised in ‘full RTW’, ‘partial RTW’, and ‘no RTW’. Subgroup analysis on the level of RTW was done for gender, age, type of injury, ISS, and MAIS. RTW was also analyzed in relation to each health domain of the EQ-5D-5L and cognition.

### Data analysis

Statistical analyses were done with Statistical Package for Social Sciences version 24.0 (SPSS, Chicago, IL).

Normality of continuous variables was tested using the Shapiro–Wilk test. All continuous variables were non-normally distributed. Descriptive statistics are presented as median (interquartile range, IQR) for continuous variables and number (percentage) for categorical variables.

A Mann–Whitney test was used for comparing two groups. A Kruskal–Wallis test was used for multiple groups, and in case of significant differences, groups were tested pairwise with Mann–Whitney tests. For nominal variables, a Chi-square test or Fisher’s exact test was used as applicable (both two-sided). A *p* value of 0.05 was considered significant.

## Results

### Study population

**Fig. 1:**
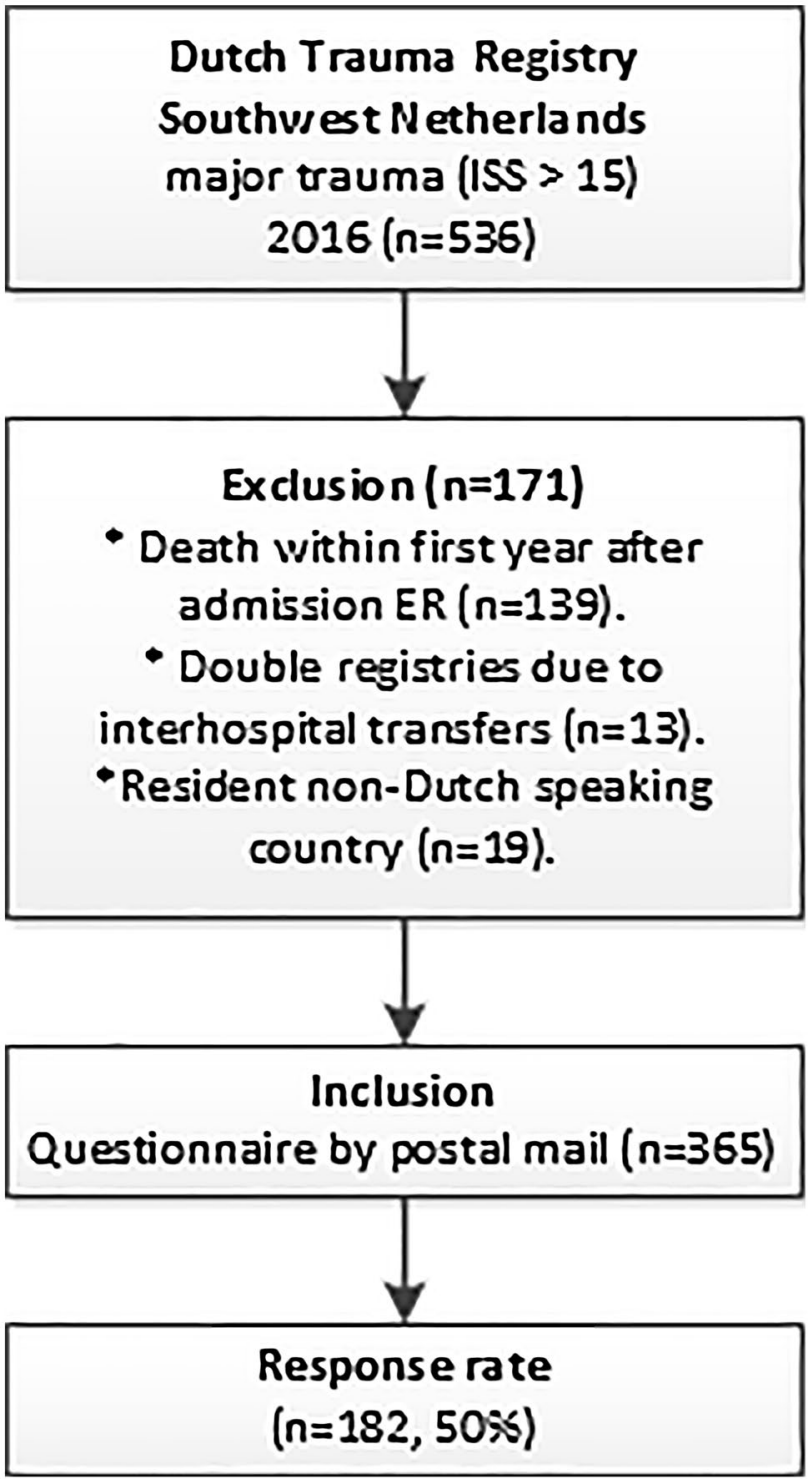
Study flowchart

Figure 1 shows the selection procedure. In 2016, 536 MT patients were registered in the DTR SW. Exclusion criteria were death within the first year (*n* = 139), double registries due to inter hospital referrals (*n* = 13), and residency abroad (*n* = 19). This resulted in a total of 369 patients who were sent questionnaires of whom 185 responded (50% response rate). The median follow-up time was 384 days (IQR 372–414). The responding group was significantly older than the non-responding group with a median age difference of little over a decade (median age 52 versus 41 years, *p* = 0.002). Overall, the responding grouped seemed representative for all included patients that were sent questionnaires (Table 1).

**Table 1:** Comparison of responders with non-responders

	Inclusion overall (*n* = 365)	Respondents (*n* = 182)	Non-respondents (*n* = 183)	*p* value
Probability of survival	0.95 (0.81–0.98)	0.94 (0.88–0.98)	0.95 (0.77–0.99)	0.554
Age at arrival ED (year)	46 (28–63)	52 (29–67)	41 (26–55)	0.002
Gender—female	124 (34%)	66 (36%)	58 (32%)	0.378
ISS ≥ 25	115 (32%)	54 (30%)	61 (33%)	0.499
ISS	21 (17–25)	20 (17–25)	21 (17–26)	0.548
Penetrating injury	8 (2.2%)	1 (0.5%)	7 (3.8%)	0.067
Endotracheal intubation prehospital	67 (18%)	29 (16%)	38 (21%)	0.279
Resuscitation prehospital	8 (2.2%)	3 (1.6%)	5 (2.7%)	0.724
Hospital LOS (days)	10 (5–19)	10 (5–17)	11 (5–21)	0.587
ICU	219 (60%)	111 (61%)	108 (59%)	0.749
Mechanical ventilation	121 (33%)	55 (30%)	66 (36%)	0.266
Surgery	183 (50%)	91 (50%)	92 (50%)	1.000
Injuries (*n*)	6 (3–9)	6 (4–9)	5 (3–9)	0.548
Fractures (*n*)	2 (1–4)	2 (1–4)	2 (1–4)	0.365
Cause—violence	12 (3%)	3 (2%)	9 (5%)	0.381
Cause—traffic	150 (41%)	79 (43%)	71 (39%)	
Cause—work	33 (9%)	19 (10%)	14 (8%)	
Cause—private	119 (33%)	56 (31%)	63 (34%)	
Cause—sports	29 (8%)	16 (9%)	13 (7%)	
Cause—self-inflicted	19 (5%)	8 (4%)	11 (6%)	

Data are reported as medians (IQR) or as *n* (%). There are no missing data

*ED* Emergency Department, *ISS* Injury Severity Score, *LOS* Length of Stay, *ICU* combination of admission to an ICU (Intensive Care Unit), High Care Unit (HCU); or Medium Care Unit (MCU)

### Overall health state, EuroQoL-5D-5L, and cognition

Eighty (44%) responders reported to be worse off than before trauma. The median EQ-US and EQ-VAS of all respondents (*n* = 182) were 0.81 (IQR 0.61–0.89) and 70 (IQR 60–80), respectively.

Limitations were prevalent in all health dimensions of the EQ-5D-5L; 90 (50%) responders reported limitations with mobility, 36 (20%) responders reported limitations with self-care, 108 (61%) responders reported limitations during daily activities, 129 (73%) responders reported limitations due to pain or discomfort, and 70 (39%) responders reported limitations with anxiety or depression. In the Dutch reference population [22], percentages of limitations in these health domains were 16.5%, 3.2%, 12.5%, 30.8%, and 10.6% respectively.

Cognitive limitations were reported by 102 (61%) responders. In the Dutch reference population [22], 7.5% reported cognitive limitations.

Table 2 shows the EQ-US, the EQ-VAS, and all health domains of the EQ-5D-5L and cognition for different subgroups. A statistically significant difference between age group 0–17 (*n* = 21) and 18–55 (*n* = 84) was present for EQ-US (0.89 versus 0.76, *p* = 0.005). Age group 0–17 (*n* = 21) scored significantly higher (*p* < 0.001) than age groups 18–55 (*n* = 84) and 55 + (*n* = 77) on their general health state (EQ-VAS 90 versus 70 and 70, respectively). Having comorbidities was associated with a worse EQ-US (healthy 0.82, one comorbidity 0.81 and > 1 comorbidities 0.65, *p* = 0.027), a worse EQ-VAS (healthy 75, one comorbidity 70 and > 1 comorbidities 68, *p* = 0.002), and more cognitive limitations (healthy 50%, one comorbidity 81% and > 1 comorbidities 63, *p* = 0.002). Educational level was linear associated with cognitive limitation (low 75%, middle 54, high 44%, *p* = 0.008).

**Table 2:** EQ-US, EQ-VAS and cognition 1 year after MT, in relation to gender, age, type of injury, ISS category, and severity of injuries per body region (MAIS)

	*n*	EQ-US (*n* = 178)	EQ-VAS (*n* = 174)	Limitations in health domains					
				Mobility (*n* = 180)	Self-care (*n* = 179)	Daily activities (*n* = 179)	Pain and discomfort (*n* = 179)	Anxiety and depression (*n* = 180)	Cognition (*n* = 168)
All respondents	182	0.81 (0.61–0.89; 4)	70 (60–80; 8)	89 (48.9; 3)	36 (19.8; 4)	108 (59.3; 4)	129 (70.9; 4)	70 (38.5; 3)	102 (56.0; 14)
Gender									
Male	116	0.79 (0.60–0.89; 4)	71 (60–82; 4)	53 (46.9; 3)	24 (21.4; 4)	69 (61.6; 4)	80 (71.4; 4)	45 (39.8; 3)	64 (60.4; 10)
Female	66	0.81 (0.60–0.89; 0)	70 (59–80; 4)	36 (54.5; 0)	12 (18.2; 0)	39 (59.1; 0)	49 (74.2; 0)	25 (37.9; 0)	38 (61.3; 4)
Age									
0–17	21	0.89 (0.75–1.00; 0)^A^	90 (76–99; 1) ^D^	1 (4.8; 0)^D^	1 (4.8; 0)	7 (33.3; 0)^C^	11 (52.4; 0)^B^	7 (33.3; 0)	12 (60.0; 1)
18–55	84	0.76 (0.56–0.85; 1)	70 (59–80; 2)	44 (53.0; 1)	15 (18.1; 1)	60 (72.3; 1)	68 (81.9; 1)	38 (45.8; 1)	50 (62.5; 4)
55 +	77	0.82 (0.54–94; 3)	70 (55–80; 5)	44 (57.9; 1)	20 (26.7; 2)	41 (54.7; 2)	50 (66.7; 2)	25 (32.9; 1)	40 (59.4; 8)
Education (age 18 +)									
Low	68	0.74 (0.49–0.87; 3)	65 (50–75; 5)	40 (60.6; 2)	18 (29.2; 3)	48 (73,8; 3)	51 (78.5; 3)	32 (48.5; 2)	46 (75.4; 7)^B^
Middle	58	0.81 (0.62–0.89; 1)	74 (60–84; 2)	29 (50.9; 1)	9 (15.8; 1)	35 (61,4; 1)	43 (75.4; 1)	22 (38.6; 1)	29 (53.7; 4)
High	29	0.83 (0.69–0.89; 0)	70 (63–80; 0)	16 (55.2; 0)	5 (17.2; 0)	15 (51.7; 0)	21 (72.4; 0)	8 (27.6; 0)	12 (44.4; 2)
Comorbidity									
None	99	0.82 (0.66–0.91; 0)^A^	75 (65–90; 5)^C^	41 (41.1; 0)^B^	16 (16.2; 0)^A^	55 (55.6; 0)	70 (70.7; 0)	34 (34.3; 0)	45 (49.5; 8)^D^
1	50	0.81 (0.57–0.92; 2)	70 (60–75; 1)	25 (52.1; 2)	7 (14.6; 2)	31 (64.6; 2)	32 (66.7; 2)	18 (37.5; 2)	39 (81.3; 2)
> 1	33	0.65 (0.41–0.87; 1)	68 (50–75; 1)	23 (69.7; 0)	13 (40.6; 1)	22 (68.8; 1)	27 (84.4; 1)	18 (54.5; 0)	19 (63.3; 3)
Injury severity									
ISS 16–24	128	0.84 (0.67–0.92; 2)^D^	75 (64–85; 7)^D^	55 (43.0; 0)^B^	22 (17.3; 1)	68 (53.5; 1)^C^	86 (67.7; 1)^A^	42 (32.8; 0)^B^	63 (53.4; 10)^C^
ISS 25 +	54	0.66 (0.34–0.84; 2)	60 (48–75; 1)	34 (65.4; 2)	14 (26.9; 2)	40 (76.9; 2)	43 (82.7; 2)	28 (53.8; 2)	39 (78.0; 4)
MAIS									
Head									
None	62	0.78 (0.48–0.89; 1)	70 (50–84; 2)	31 (50.8; 1)	13 (21.3; 1)	35 (57.4; 1)	49 (80.3; 1)	25 (41.0; 1)	31 (51.7; 2)^D^
1–2	27	0.81 (0.60–0.90; 2)	70 (60–83; 1)	15 (57.7; 1)	7 (28.0; 2)	17 (68.0; 2)	19 (76.0; 2)	9 (34.6; 1)	8 (36.4; 5)
3 +	93	0.81 (0.61–0.92; 1)	72 (60–80; 5)	43 (46.2; 0)	16 (17.2; 0)	56 (60.2; 0)	61 (65.6; 0)	36 (38.7; 0)	64 (73.6; 6)
Face									
None	124	0.81 (0.60–0.89; 3)	71 (55–85; 5)	60 (49.2; 2)	23 (19.0; 3)	70 (57.9; 3)	90 (74.4; 3)	49 (40.2; 2)	62 (53.9; 8)^A^
1–2	53	0.79 (0.57–0.91; 1)	71 (60–80; 3)	25 (47.2; 0)	12 (22.6; 0)	34 64.2(; 0)	34 (64.2; 0)	18 (34.0; 0)	36 (73.5; 4)
3 +	5	0.74 (0.44–0.82; 0)	70 (53–73; 0)	4 (80.0; 0)	1 (20.0; 0)	4 (80.0; 0)	5 (100.0; 0)	3 (60.0; 0)	5 (100.0; 0)
Neck									
None	176	0.81 (0.60–0.89; 4)	71 (60–82; 8)	86 (49.4; 2)	34 (19.7; 3)	102 (59.0; 3)	123 (71.1; 3)	64 (36.8; 2) ^B^	99 (60.7; 13)
1–2	1	0.74 (0.74–0.74; 0)	65 (65–65; 0)	1 (100.0; 0)	0 (0.0; 0)	1 (100.0; 0)	1 (100.0; 0)	1 (100.0; 0)	0 (0.0; 0)
3 +	5	0.64 (0.59–0.77; 0)	67 (63–73; 0)	2 (40.0; 0)	2 (40.0; 0)	5 (100.0; 0)	5 (100.0; 0)	5 (100.0; 0)	4 (80.0; 0)
Thorax									
None	92	0.81 (0.53–0.92; 0)	75 (60–85; 4)	46 (50.0; 0)	22 (23.9;)	53 (57.6; 0)	63 (68.5; 0)	30 (32.6; 0)	53 (61.6; 6)
1–2	21	0.74 (0.46–0.85; 0)	70 (58–83; 0)	12 (57.1; 0)	4 (19.0; 0)	14 (66.7; 0)	16 (76.2; 0)	11 (52.4; 0)	11 (57.9; 2)
3 +	69	0.82 (0.64–0.89; 4)	70 (58–80; 4)	31 (46.3; 2)	10 (15.2; 3)	41 (62.1; 3)	50 (75.8; 3)	29 (43.3; 2)	39 (60.9; 5)
Abdomen									
None	150	0.81 (0.61–0.89; 2)	71 (60–80; 6)	75 (50.3; 1)	32 (21.5; 1)	92 (61.7; 1)	106 (71.1; 1)	53 (35.6; 1)	88 (62.4; 9)
1–2	14	0.82 (0.61–0.94; 1)	80 (45–90; 1)	6 (42.9; 0)	1 (7.7; 1)	7 (53.8; 1)	10 (76.9; 1)	8 (57.1; 0)	6 (50.0; 2)
3 +	18	0.72 (0.24–0.94; 1)	65 (50–80; 1)	8 (47.1; 1)	3 (17.6; 1)	9 (52.9; 1)	13 (76.5; 1)	9 (52.9; 1)	9 (56.3; 2)
Spine									
None	130	0.82 (0.65–0.92; 1)^A^	71 (60–81; 6)	57 (43.5; 0)^A^	22 (16.9; 0)	72 (55.4; 0)	86 (66.2; 0)^A^	49 (37.4; 0)	79 (64.2; 7)
1–2	25	0.75 (0.39–0.85; 2)	70 (55–82; 2)	15 (65.2; 2)	6 (26.1; 2)	16 (69.6; 2)	20 (87.0; 2)	10 (43.5; 2)	13 (61.9; 4)
3 +	27	0.73 (0.46–0.85; 1)	70 (60–80; 0)	17 (65.4; 1)	8 (30.8; 1)	20 (76.9; 1)	23 (88.5; 1)	11 (42.3; 1)	11 (44.0; 2)
Upper extremity									
None	106	0.81 (0.59–0.92; 0)	70 (60–85; 1)	55 (51.9; 0)	21 (19.8; 0)^A^	58 (54.7; 0)	71 (67.0; 0)	39 (36.8; 0)	60 (60.6; 16)
1–2	74	0.80 (0.60–0.89; 4)	70 (58–80; 7)	33 (45.8; 2)	13 (18.3; 3)	48 (67.6; 3)	56 (78.9; 3)	30 (41.7; 2)	42 (61.8; 6)
3 +	2	0.71 (0.66–0.71; 0)	65 (50–65; 0)	1 (50.0; 0)	2 (100.0; 0)	2 (100.0; 0)	2 (100.0; 0)	1 (50.0; 0)	1 (50.0; 0)
Lower extremity									
None	115	0.82 (0.61–1.00; 2) ^A^	75 (60–85; 5)	48 (41.7; 0)^D^	23 (20.2; 1)	61 (53.5; 0)^A^	72 (63.2; 0)^C^	42 (36.5; 0)	62 (57.4; 7)
1–2	34	0.82 (0.66–0.86; 1)	70 (65–82; 2)	16 (48.5; 1)	4 (12.1; 1)	22 (66.7; 1)	29 (87.9; 1)	12 (36.4; 1)	20 (69.0; 5)
3 +	33	0.66 (0.30–0.84; 1)	60 (50–79; 1)	25 (78.1; 1)	9 (28.1; 1)	25 (78.1; 1)	28 (87.5; 1)	16 (50.0; 1)	21 (65.6; 1)
External									
None	159	0.81 (0.61–0.89; 4)	71 (60–82; 8)	77 (49.0; 2)	30 (19.2; 3)	97 (62.2; 3)	110 (70.5; 3)	58 (36.9; 2)	86 (58.1; 11)
1–2	17	0.76 (0.51–0.89; 0)	70 (48–83; 0)	9 (52.9; 0)	4 (23.5; 0)	9 (52.9; 0)	14 (82.4; 0)	9 (52.9; 0)	11 (73.3; 2)
3 +	6	0.76 (0.53–0.90; 0)	60 (39–80; 0)	3 (50.0; 0)	2 (33.3; 0)	2 (33.3; 0)	5 (83.3; 0)	3 (50.0; 0)	6 (100.0; 0)

Data are reported as medians (IQR; no. missing values) or as *n* (%; no. missing values)

*ISS* Injury Severity Score, *MAIS* Maximum Abbreviated Injury Score

^A^0.05 ≤ *p* < 0.01

^B^0.01 ≤ *p* < 0.005

^C^0.005 ≤ *p* < 0.001

^D^*p* ≤ 0.001

Patients with an ISS ≥ 25 (*n* = 54) scored significantly lower than patients with ISS 16–24 (*n* = 128) on EQ-US and EQ-VAS with a median utility sum score of 0.66 and 0.84, respectively (*p* < 0.001), and a median VAS of 60 and 75, respectively (*p* < 0.001). Patients with ISS ≥ 25 (*n* = 54) reported cognitive limitations (*n* = 39, 78.0%) significantly (*p* = 0.003) more often than patients with ISS 16–24 (*n* = 63, 53.4%).

Patients with a severe lower extremity injury (MAIS ≥ 3) had significantly lower overall scores (*n* = 33, EQ-US = 0.66, EQ-VAS = 60) than patients with moderate (MAIS 1–2) lower extremity injuries (*n* = 34, EQ-US 0.82, *p* = 0.048) or patients without lower extremity injuries (*n* = 115, EQ-US = 0.82, *p* = 0.006; EQ-VAS = 75, *p* = 0.024). Patients with severe spine injuries (MAIS ≥ 3) scored significantly worse on EQ-US than patients without spine injuries (*n* = 27, EQ-US = 0.73 versus *n* = 130, 0.82, *p* = 0.05). Patients with severe head injuries (MAIS ≥ 3, *n* = 63, 73%) had significantly more often cognitive limitations than patients with moderate (MAIS 1–2, *n* = 8, 36%, *p* = 0.001) or no head injuries (*n* = 31, 52%, *p* = 0.008). Patients with moderate (MAIS 1–2, *n* = 35, 73%) and severe face injuries (MAIS ≥ 3, *n* = 5, 100%) reported significantly (*p* = 0.043 and *p* = 0.025 respectively) more often cognitive limitations than patients with no injuries in the face (*n* = 62, 53%).

### Return to work

Although 103 of the 185 respondents stated that they had paid work before their trauma, complete data were acquired of 100 patients which could be analyzed as working population. One year after trauma, overall RTW rate was 68% (*n* = 68), 37% (*n* = 37) worked as much or more as before trauma, and 31% (*n* = 31) partially resumed work (Table 3). Patients who started working partially again worked 14 (IQR 7–28) h per week less than before.

**Table 3:** RTW 1 year after MT, in relation to gender, age, type of injury, ISS category, and maximum AIS per body region (MAIS)

	Overall (*n* = 100)*	Level of return to work			
		Fully (*n* = 37)	Partially (*n* = 31)	No return (*n* = 32)	*p* value
Female	28 (28%)	7 (19%)	12 (39%)	9 (28%)	0.194
Age 18–55	69 (71%)	25 (36%)	22 (32%)	22 (32%)	0.912
Education					
Low	39 (40%)	10 (27%)	13 (42%)	16 (53%)	0.107
Middle	39 (40%)	16 (43%)	11 (36%)	12 (40%)	
High	20 (20%)	11 (30%)	7 (23%)	2 (7%)	
Comorbidity					
None	62 (62%)	28 (76%)	16 (52%)	18 (56%)	0.297
1	26 (26%)	6 (16%)	10 (32%)	10 (31%)	
> 1	12 (12%)	3 (8%)	5 (16%)	4 (13%)	
ISS 16–24	68 (68%)	30 (81%)	22 (71%)	16 (50%)	0.020
MAIS					
Head					
None	35 (35%)	11 (30%)	12 (39%)	12 (38%)	0.815
1–2	18 (18%)	8 (22%)	6 (19%)	4 (13%)	
3 +	47 (47%)	18 (49%)	13 (42%)	16 (50%)	
Face					
None	69 (69%)	26 (70%)	19 (61%)	24 (75%)	0.674
1–2	26 (26%)	10 (27%)	9 (29%)	7 (22%)	
3 +	5 (5%)	1 (3%)	3 (10%)	1 (3%)	
Neck					
None	96 (96%)	36 (97%)	30 (97%)	30 (94%)	0.432
1–2	1 (1%)	–	1 (3%)	–	
3 +	3 (3%)	1 (3%)	–	2 (6%)	
Thorax					
None	41 (41%)	18 (49%)	10 (32%)	13 (41%)	0.338
1–2	15 (15%)	3 (8%)	8 (26%)	4 (13%)	
3 +	44 (44%)	16 (43%)	13 (42%)	15 (47%)	
Abdomen					
None	82 (82%)	30 (81%)	26 (84%)	26 (81%)	0.479
1–2	8 (8%)	5 (14%)	1 (3%)	2 (6%)	
3 +	10 (10%)	2 (5%)	4 (13%)	4 (13%)	
Spine					
None	64 (64%)	23 (62%)	21 (68%)	20 (63%)	0.282
1–2	18 (18%)	9 (24%)	2 (7%)	7 (22%	
3 +	18 (18%)	5 (14%)	8 (26%)	5 (16%)	
Upper extremity					
None	58 (58%)	22 (60%)	19 (61%)	17 (53%)	0.819
1–2	40 (40%)	15 (41%)	11 (36%)	14 (44%)	
3 +	2 (2%)	–	1 (3%)	1 (3%)	
Lower extremity					
None	60 (60%)	23 (62%)	18 (58%)	19 (60%)	0.881
1–2	20 (20%)	8 (22%)	7 (23%)	5 (16%)	
3 +	20 (20%)	6 (16%)	6 (20%)	8 (25%)	
External					
None	87 (87%)	35 (95%)	28 (90%)	24 (75%)	0.138
1–2	10 (10%)	2 (5%)	2 (7%)	6 (19%)	
3 +	3 (3%)	–	1 (3%)	2 (6%)	

Data are reported as *n* (%)

*ISS* Injury Severity Score, *MAIS* Maximum Abbreviated Injury Score

*Three responders reported work, but did not state how many hours and were left out of the analysis

**Fig. 2:**
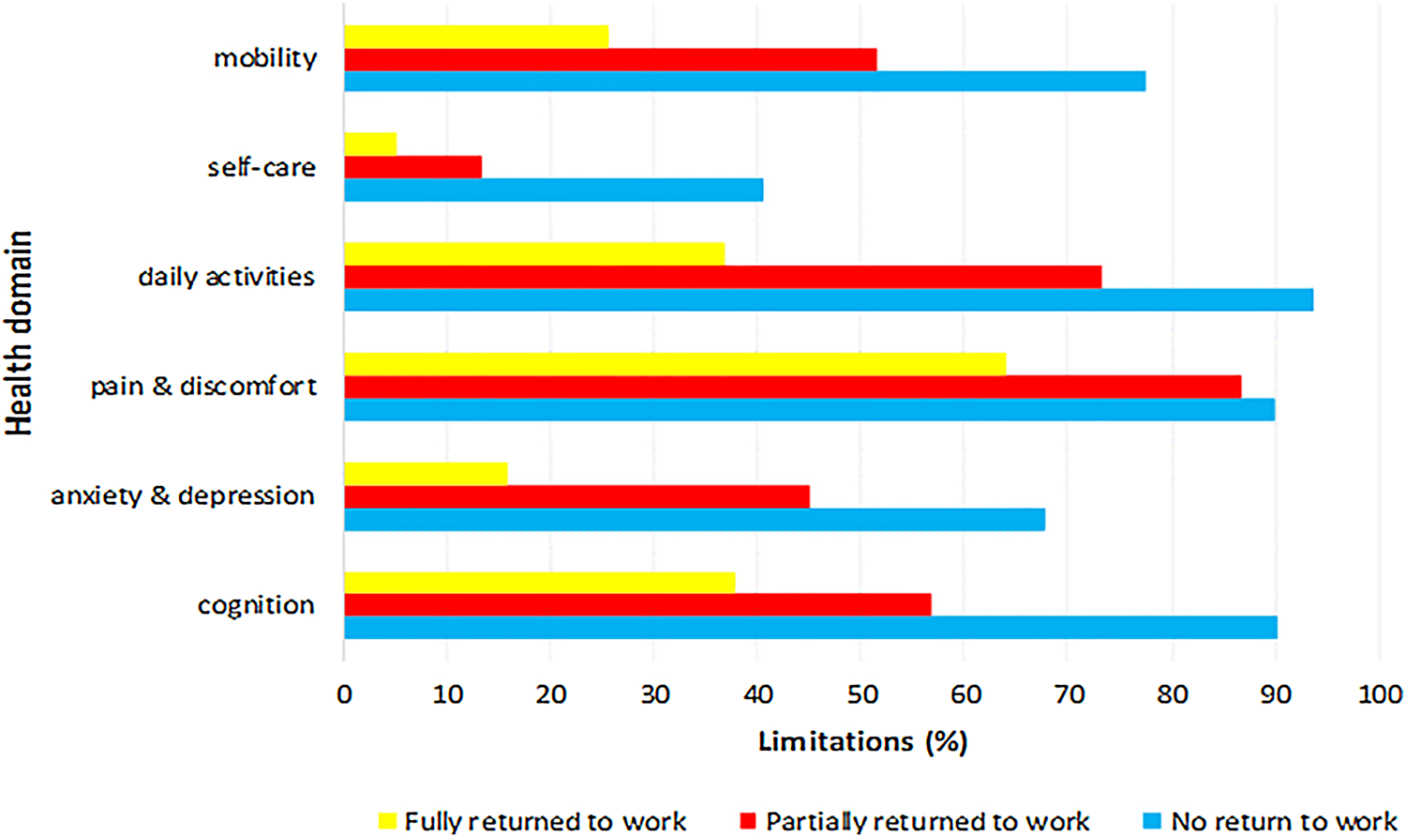
Limitations (%) per health domain of the EQ-5D-5L and cognition per return to work group

All health domains including cognition are displayed per RTW group in Fig. 2. Patients who worked fully again (*n* = 37) had a median (IQR) EQ-US and EQ-VAS of 0.89 (0.82–1.00) and 80 (70–90), respectively. This differed significantly with the partial working group (*n* = 31, EQ-US 0.77 (0.66–0.85), *p* < 0.001; EQ-VAS 70 (62–80), *p* = 0.001) and the unemployed group (*n* = 32, EQ-US 0.49 (0.23–0.69), *p* < 0.001; EQ-VAS 55 (40–72), *p* < 0.001). Partial and no RTW also differed significantly on the EQ-US (*p* < 0.001) and EQ-VAS (*p* = 0.002). Comparing RTW status per health domain gave significant results for all health domains and cognition. A better RTW status was associated with better scores on all health domains of the EQ-5D-5L and cognition. Patients who returned fully to work could still experience limitations in health domains (from 5% (*n* = 2) in self-care, up to 65% (*n* = 24) in pain or discomfort).

In Table 3, fully, partial, and no RTW are shown for different subgroups. Patients with less severe injuries (ISS 16–24, *n* = 68) returned to work more often (*p* = 0.020). Level of work resumption was unrelated to gender, type of injury, age, level of education, no. of comorbidities, and (severity) of specific organ injuries 1 year after MT.

## Discussion

This study assessed functional limitations by the means of health-related QoL and RTW in patients 1 year after MT (ISS > 15) from a trauma network perspective. The median overall utility score was 0.81 and the median general health state 70. Over 60% reported cognitive problems and less than 40% fully returned to work. If patients reported limitations, the majority scored little or moderate problems on all health domains. Severe problems or ‘not able’ was scored less (4.4–20.7%). Limitations within the five health domains of the EQ-5D or cognition were reported by 87% of all responders. In contrast, 44% of the respondents reported to be worse off after their trauma. Many respondents might have had functional limitations before their trauma. Trauma populations are associated with higher pre-existing morbidity, compared with non-injured populations, which might be prone to an overestimation of problems post-injury [24]. In addition, recall bias could have caused responders to overconfidently report their overall health state.

A mean overall utility score of 0.88 has been reported for the Dutch population [22]. Dutch non-hospitalized trauma patients have a comparable health status 5 months after trauma (0.87) [25]. A mean overall utility score of ≈ 0.84 has been reported for a Dutch hospitalized trauma population 1 year after trauma (taken from graph) [26]. For the majority of the respondents in the present study, QoL was below population norms 1 year after MT.

Dutch studies [27–29] comparable to the current study were done on adult MT populations in a single-level I trauma center with the usage of EQ-5D-3L. A mean EQ-US of 0.69 was reported by two studies [27, 28] and a median of 0.73 in the remaining study [29]. A recent regional study [30] reported a mean overall utility score with EQ-5D-3L on adults of 0.77 1 year after MT trauma. The adult population in the current study had a median EQ-US of 0.81 (IQR 0.61–0.89), and for comparing purposes a mean of 0.70 (SD 0.30). Prevalence of limitations in most health domains remains high and yields a similar pattern compared to previous mentioned single-center studies. Despite improvements in trauma care, health domains mobility, daily activities, and pain or discomfort even display increased prevalence of limitations. Future research should focus on interventions that reduce common chronic complaints that impact QoL after MT. Such interventions should not only be addressed to hospitals, but in collaboration with revalidation trajectories on all levels. Prevention of trauma by wearing helmets in traffic by people on (motor driven) bicycles, separating cycle roads from major roads, fall prevention for the elderly, and reducing substance abuse and psychiatric disorders are some examples of favorable strategies over cure and care.

A straightforward comparison of the present study with international publications on (health-related) QoL after MT is difficult due to the use of different questionnaires, reporting the same instruments differently, variability in inclusion criteria, and the usage of different AIS revisions for injury coding. Studies that used unknown or older AIS revisions reported a mean EQ-US range of 0.60–0.69 or a median EQ-US range of 0.60–0.73, within a response time range of 6–18 months after trauma. The current study combined injury coding and inclusion on the basis of AIS08 with the EQ-5D-5L. Only one MT study [13] with AIS08 injury coding that reported QoL and had general inclusion criteria used the 5L version (median EQ-US ≈ 0.80, taken from graph). MT patients have higher mortality rates and more frequently need intensive care and urgent surgery when the ISS is derived from AIS08 compared to older AIS revisions [31]. One would also expect a worse QoL compared to previous studies with injury coding on the basis of AIS98; however, this was not the case. Two factors could have been a major contributor to better QoL in the current cohort. First, the EQ-5D with a five-level answer option might have had a moderating effect compared to the three-level version. Second, (major) trauma networks have matured.

Especially, international studies handling QoL after MT from a regional trauma network perspective are scarce [7, 9, 10]. Most studies on QoL of MT patients included adult populations admitted to a single TC. In 2015–2018, around one-third of all MT patients in the DTR SW cohort were directly admitted to NTC’s. The current study looked at all MT patients captured in one TC and 11 NTCs, a truly representative national sample.

Patients with an ISS ≥ 25 scored worse on EQ-US, EQ-VAS, and cognition than patients with an ISS 16–24. A similar association was found between RTW and ISS. These results suggest that being able to return to work has a positive impact on the perceived QoL 1 year after MT, or that improved QoL may facilitate RTW. QoL and RTW are associated. Even though RTW can indicate a certain health status, the question is whether it is a reliable parameter to evaluate trauma outcome, since it can be affected by many other factors (e.g., economic) [32].

### Limitations

First, a response rate of 50% might have resulted in a non-response bias. A part of the patients will not have been able to respond due to (severe) problems with, e.g., hand writing, post-traumatic stress, depression, or level of consciousness. MT patients that have had fewer problems post-injury might have been more likely to respond, because they have fewer attention consuming activities around their recovery or found participating not that confronting. These biases will result in an overestimation of the recovery status of the studied cohort 1 year after MT. However, during reminder calls, statements on not participating due to no experienced problems were also not uncommon. Recall bias could have resulted in an underestimation of the overall health state of the responders. In general, reliable information on pre-injury is lacking in this study. Such information is appropriate in compensating for attribution bias.

Second, the EuroQOL-group advises a proxy version for ages 0–7, the EQ-5D-Y for ages 8–11, and for ages 12–18, the adult version can be used (for ages 12–15, the EQ-5D-Y is recommended). These versions are developed on the basis of the EQ-5D-3L, and at present, a utility value set for the EQ-5D-Y is lacking. In the present study, the adult version of the EQ-5D-5L was used for children, which probably resulted in an overestimation of the QOL of children aged 15 years and younger [33].

Third, the definition of MT has always been under the debate, we used a threshold of ISS > 15. The ISS was calculated following AIS08, while the most recent version being AIS15. It is unclear what the effects of the AIS15 are on an MT cohort with an ISS > 15. In addition, a certain part of injuries are wrongly coded due to inter-rater agreement and reliability limitations of the AIS [34]. Adding patients to the current cohort with a significant trauma mechanism but an ISS < 16 in combination with AIS15 could have created a more comprehensive and contemporary overview of QoL after MT.

The extent of problems people experience 1 year after MT stresses the necessity of a long multicenter follow-up. More insight can be gained in the long-term recovery status of MT patients. Physical and mental recovery take a long time, but social integration might take much longer; especially when not just considering the effect of traumatic events on individuals, but also on their social environment and relatives.

## Conclusion

One year after major trauma, the majority of patients experience problems in all health domains of the EQ-5D-5L and cognition, and function below population norms. Trauma care should focus on these health domains in collaboration with other revalidation disciplines. Return to work status was associated with all health domains of the EQ-5D-5L and cognition, showing that focus on health status of trauma patients in recovery trajectories can potentially have many positive effects.

## Data Availability

Any reasonable request for data will be taken into consideration; however, data are not public due to the GDPR.

## Code availability

If data will be shared, software will be made available as well.

## Declarations

### Disclaimer

The views expressed in the submitted article are of the authors and not of the represented institutions.
